# Synergistic antibiotic effect of looped antimicrobial peptide CLP-19 with bactericidal and bacteriostatic agents

**DOI:** 10.18632/oncotarget.18124

**Published:** 2017-05-23

**Authors:** Di Li, Ya Yang, Zhiqiang Tian, Jun Lv, Fengjun Sun, Qian Wang, Yao Liu, Peiyuan Xia

**Affiliations:** ^1^ Department of Pharmacy, Southwest Hospital, Third Military Medical University, Chongqing, China; ^2^ Department of Pharmacy, Second Affiliated Hospital, Chongqing Medical University, Chongqing, China; ^3^ Department of Immunology, Third Military Medical University, Chongqing, China

**Keywords:** antimicrobial peptides, CLP-19, synergistic effect, hydroxyl radicals, LPS, Immunology and Microbiology Section, Immune response, Immunity

## Abstract

The treatment of drug-resistant infections is complicated and the alarming rise in infectious diseases poses a unique challenge for development of effective therapeutic strategies. Antibiotic-induced liberation of the bacterial endotoxin lipopolysaccharide (LPS) may have immediate adverse effects promoting septic shock in patients. In the present study, we first confirmed our previous finding that looped antimicrobial peptide CLP-19 exerts non-specific direct antibacterial activity with no toxic to mammalian cells and second revealed that CLP-19 has synergistic effect to enhance the antibacterial activities of other conventional bactericidal (ampicillin and ceftazidime) and bacteriostatic (erythromycin and levofloxacin) agents. Third, the underlying mechanism of antibiotic effect was likely associated with stimulation of hydroxyl radical generation. Lastly, CLP-19 was shown to effectively reduce the antibiotic-induced liberation of LPS, through direct neutralization of the LPS. Thus, CLP-19 is a potential therapeutic agent for combinatorial antibiotic therapy.

## INTRODUCTION

The rapid emergence of drug-resistant bacteria endangers the efficacy of antibiotics, which presents a unique and alarming challenge to clinical care [1]. Adequate antibiotic therapy is the cornerstone of appropriate management of bacteria strains and crucial for halting the evolution of bacteria with new resistance capabilities. However, therapeutic intervention based on the conventional antibiotics alone is insufficient and possibly harmful since their use can promote the pathophysiological process of septic shock. Although the pathophysiology of septic shock is not entirely understood, it is likely due to liberation of endotoxins from the bacterial cell wall during destruction of the microorganism [2]. To overcome this obstacle, various adjuvant treatment approaches have been carefully analyzed, ranging from standard intravenous immunoglobulins or endotoxin core-specific antibodies to treatment with cytokines, cytokine receptor antagonists, or immunomodulators; the results, however, have been largely disappointing. Therefore development of a novel adjuvant treatment that not only exhibits synergistic effect in combination with existing antibacterial agents but that also relieves the excessive endotoxin-induced septic shock is a critical step in the battle against this serious threat to public health.

Antimicrobial peptides (AMPs), isolated from a wide range of species (e.g. amphibians, fish, mollusca, insects, mammals, and plants, *etc*.), act in host defense against pathological inflammation and microbial infections. Immunomodulators such aspolymyxin B [3], bactericidal/permeability-increasing protein [4, 5], CAP-18 [6, 7], and mastoparan peptide [8] may protect against lethal inflammatory response. On the other hand, ranalexin [9, 10], OH-CATH [11] and arenicin-1 peptide [12] may kill bacteria (both Gram-negative and Gram-positive), viruses, fungi, protozoa, and even cancerous cells. In addition, when used against many kinds of bacterial infections, arenicin-1 [12], ranalexin [13] and P5-18mer [13] have been shown to exert synergistic antibiotic effects.

We previously characterized the core domain of *Limulus* anti-LPS factor (LALF; amino acids 31-52), a small basic protein derived from the arthropods *Tachypleus tridentatus* and *Limulus polyphemus*, to generate a novel peptide, CLP-19. Composed of 19 amino acid residues, CLP-19 is head-to-tail looped *via* a disulfide bond and possesses cationic, amphipathic structure. The inherent potency of CLP-19 was shown to not only involve direct antibacterial activity against various pathogenic bacteria but also exert a robust anti-LPS activity that prevents the subsequent stimulation of the innate immune system activator, TLR4, as well as the successive induction of cytokines production and release [14–16].

In the present study, we sought to investigate whether co-treatments with CLP-19 and other antibiotics have the synergistic effect against bacterial growth and elucidate the underlying mechanism.

## RESULTS

### CLP-19 displays non-selective direct antibacterial activity as compared to other conventional antibiotics

In this assay, the minimum inhibitory concentrations (MICs) of CLP-19, ampicillin, ceftazidime, erythromycin, levofloxacin and S-LALF peptide were determined. The sensitivity of bacteria to the peptides and antibiotics is presented in Table 1. Ampicillin showed antibacterial activity against *E. coli* and *S. aureus* at MIC values of 4 μg/mL and 2 μg/mL but showed no influence on the survival of *A. baumannii* and *P. aeruginosa*, even up to 256 μg/mL MIC. The MIC of ceftazidime against Gram-negative and Gram-positive bacteria ranged from 0.25 μg/mL to 16 μg/mL. Erythromycin exhibited antibacterial activity against *S. aureus* at the MIC value of 1 μg/mL but showed no effect on other microbes tested, even with the highest MIC tested. The MICs of levofloxacin against *E. coli*, *S. aureus* and *P. aeruginosa* were relatively low (0.06 μg/mL, 0.5 μg/mL and 4 μg/mL respectively), yet high ( > 256 μg/mL) against *A. baumannii*. It is worth noting that CLP-19 showed antibacterial activity at MICs ranging from 16 ug/mL to 32 μg/mL against *E. coli*, *S. aureus* and *A. baumannii*, suggesting non-selective antibacterial activity for Gram-negative and Gram-positive bacteria. However, CLP-19 showed no observable antibacterial activity against *P. aeruginosa* ( > 256 μg/mL). S-LALF, a precursor peptide of CLP-19, showed no antibacterial activity against any of the above mentioned bacteria.

**Table 1:** MICs of CLP-19, ampicillin, ceftazidime, erythromycin and levofloxacin against *E. coli, S. aureus, A. baumannii and P. aeruginosa*

Bacterial strains	MIC, μg/mL					
	Ampicillin	Ceftazidime	Erythromycin	Levofloxacin	CLP-19	S-LALF
*E. coli* (ATCC 25922)	4	0.25	> 256	0.06	16	> 256
*S. aureus* (ATCC 29213)	2	16	1	0.5	16	> 256
*A. baumannii* (ATCC 19606)	> 256	4	> 256	> 256	32	> 256
*P. aeruginosa* (ATCC 27853)	> 256	2	> 256	4	> 256	> 256

Bacterial strains at mid-log phase (1×10^6^/mL) were treated with increasing concentrations of antibiotic agents with incubation at 37°C for 18 h. Growth was assayed by monitoring OD_620_. S-LALF served as control. (*n* = 3).

### The therapeutic doses of CLP-19 show minimal cytotoxicity

To evaluate the toxicity of CLP-19 *in vitro*, the hemolysis assay and mammalian cell toxicity assay were carried out. CLP-19 treatment at 128 μg/mL or lower did not produce any observable hemolysis or toxicity in erythrocytes and Vero cells; however, further increase of the peptide concentration, up to 256 μg/mL, produced significant cytotoxicity (Table 2). Reassuringly, the concentrations of CLP-19 required for effective antibacterial activity are much less than those that showed significant cytotoxicity to the erythrocytes and Vero cells.

**Table 2:** Toxicity of CLP-19

Concentration of CLP-19	Haemolysis, %	Reduction in cell viability, %
16	−1.16 ± 0.65	2.57 ± 3.43
32	0.08 ± 0.76	2.04 ± 1.60
64	−0.24 ± 1.32	−1.38 ± 2.27
128	0.68 ± 1.05	3.39 ± 1.44
256	38.71 ± 10.05	45.53 ± 17.52
512	72.35 ± 17.50	91.23 ± 30.71

Defibrinated horse blood and Vero cells were treated with CLP-19 for 4 h and 48 h respectively. PBS and 1% sodium dodecyl sulfate were served as negative and positive controls. The negative value indicates that haemolysis was less than the PBS control and Vero cell viability was greater than the PBS control. Numerical data represent means ± SD (*n* = 6).

### CLP-19 has synergistic antibacterial effect when applied in combination with other conventional antibiotics

The synergistic effect of CLP-19 was evaluated by determining the fractional inhibitory concentration index (FICI). Table 3 shows that the average FICI of CLP-19 ranged from 0.375 to 0.5 when used in combination with ampicillin, ceftazidime or levofloxacin, indicating that CLP-19 has a synergistic antibacterial effect when combining with these conventional antibiotics. Nevertheless, CLP-19 only showed a partial synergistic effect when used in combination with erythromycin (FICI = 0.75) against *S. aureus*. Since the MICs of ampicillin against *A. baumannii* and *P. aeruginosa*, erythromycin against *E. coli, A. baumannii* and *P. aeruginosa*, levofloxacin against *A. baumannii* and CLP-19 against *P. aeruginosa* were not obtained because of overcoming the test concentrations, the FICIs of above mentioned compounds were not able to calculate.

**Table 3:** FICIs of CLP-19 in combination with ampicillin, ceftazidime, erythromycin or levofloxacin

Bacterial strains	Ampicillin+CLP-19		Ceftazidime+CLP-19		Erythromycin+CLP-19		Levofloxacin+CLP-19	
	FICI	Interactive category	FICI	Interactive category	FICI	Interactive category	FICI	Interactive category
*E. coli* (ATCC 25922)	0.375	S	0.5	S	\	\	0.5	S
*S. aureus* (ATCC 29213)	0. 5	S	0.5	S	0.75	PS	0.5	S
*A. baumannii* (ATCC 19606)	\	\	0.5	S	\	\	\	\

A 2-dimensional checkerboard with 2-fold dilutions of each agent was set up. The FICI was calculated according to the equation: FICA + FICB = (MIC_Drug A_ in combination/MIC_Drug A_ alone) + (MIC_Drug B_ in combination/MIC_Drug B_ alone). FICI, calculated as the sum of each FIC, was interpreted as follows: FICI < 0.5, synergy; 0.5 ≤ FICI < 1, partial synergy; 1 ≤ FICI < 4, additive effect or indifference; 4 ≤ FICI antagonism. S denotes synergy and PS denotes partial synergy. (*n* = 3).

### Synergistic characteristics of CLP-19 with the conventional antibiotics

**Figure 1:**
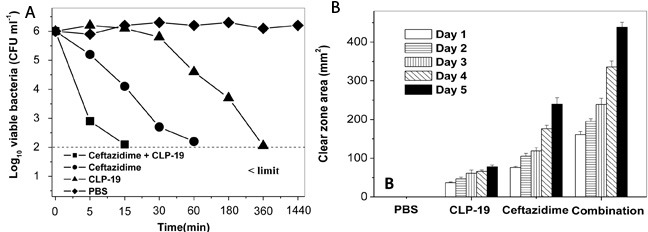
Synergistic characteristics of CLP-19 and the conventional antibiotics **A.** *E. coli* strains at mid-log phase (1×10^6^/mL) were treated with CLP-19 (16 μg/mL), ceftazidime (0.25 μg/mL) or in combination by incubating at 25°C for 5 min, 15 min, 30 min, 1 h, 3 h, 6 h and 24 h. Cells treated with PBS served as controls. Numerical data represent mean ± SD (*n* = 3). **B.** Sterile paper discs impregnated with MICs of CLP-19 alone, ceftazidime alone or in combination were placed onto the surface of a TSA plate that had been seeded with an *E. coli* suspension (1×10^8^/mL) and incubated at 37°C for 5 days, with measurement of clear zones around each disc taken every 24 h. Discs impregnated with PBS served as controls. Numerical data represent mean ± SD (*n* = 3).

To investigate the synergistic antimicrobial properties of CLP-19, the killing kinetics of CLP-19 alone, ceftazidime alone, and in combination were determined. The time-killing curves suggested that treatment of CLP-19 or ceftazidime alone for 60 or 360 min completely eliminated *E. coli*. Moreover, cells co-treated with CLP-19 and ceftazidime showed no viable bacteria within 15 min. These findings were significantly different from the control group with PBS treatment showing minimal to no change in bacterial viability over the 24 h experimental period (Figure 1A).

Moreover, stability of the antibacterial activity was determined by using disc diffusion assay. Discs containing the MIC of CLP-19 for *E. coli* caused a 36.8 ± 2.0 mm^2^ (mean ± SD) clear zone area on day 1, and this zone slightly enlarged daily until 77.9 ± 4.3 mm^2^ on day 5. The MIC of ceftazidime caused the clear zone area to increase from 76.1 ± 2.9 mm^2^ on day 1 to 239.6 ± 16.7 mm^2^ on day 5. The clear zone area of CLP-19 combined with ceftazidime was remarkably higher, being 161.0 ± 8.3 mm^2^ on day 1 and reaching 438.2 ± 13.0 mm^2^ on day 5. These results suggested that the antibacterial compounds were active during the course of the experiment (Figure 1B). The daily increase in clear zone area caused by the compounds in combination was significantly greater (*p* < 0.05) than the sum of the clear zones caused by either of the compounds individually. The similar phenomena were observed against *S. aureus* and *A. baumannii* (data not shown), indicating that the combination treatment has the synergistic antibacterial activity.

### CLP-19 or CLP-19-based combination treatments induced formation of hydroxyl radicals

**Figure 2:**
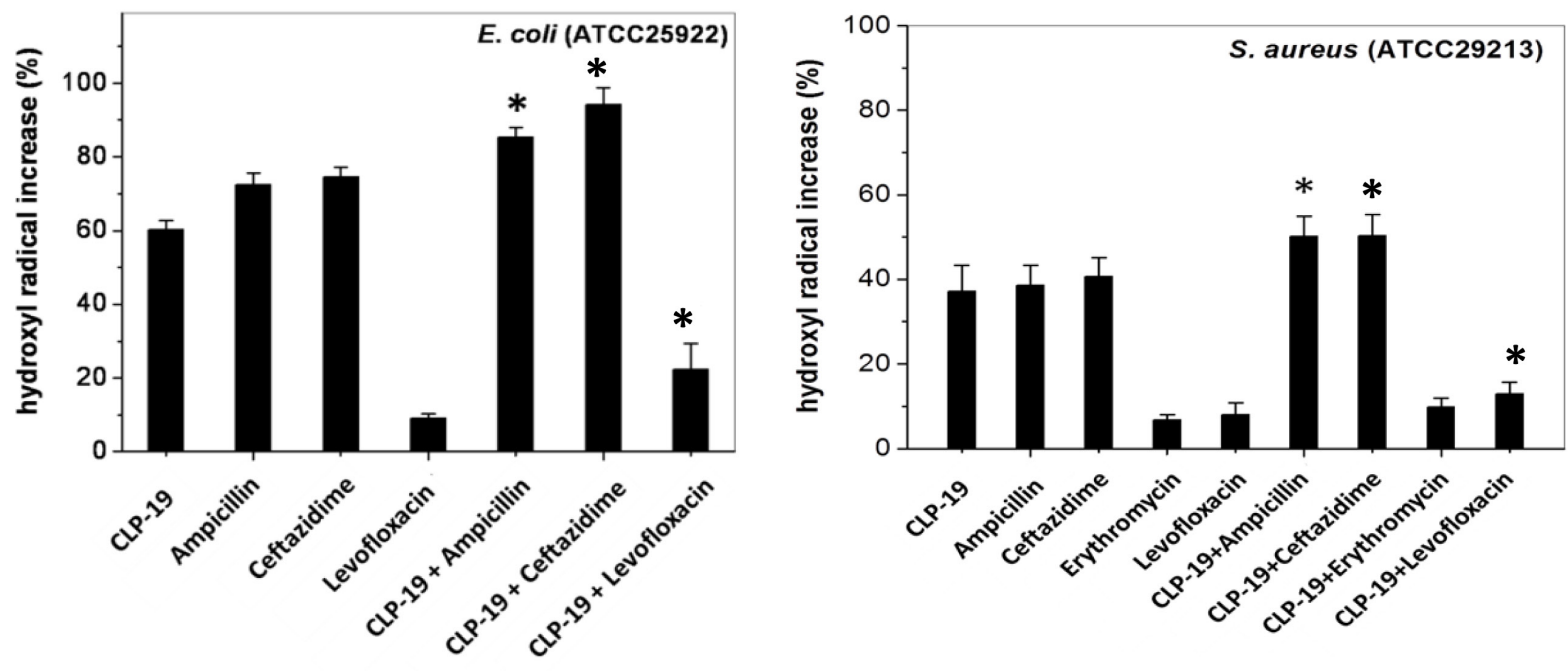
Formation of hydroxyl radicals induced by CLP-19 or CLP-19-based combination Bacteria strains at mid-log phase (1×10^6^/mL) were treated with MICs of CLP-19 alone or ampicillin, ceftazidime, erythromycin or levofloxacin alone or in combination with CLP-19 by incubating at 37°C for 2 h. Cells treated with PBS served as controls. Numerical data represent mean ± SD (*n* = 3). *p < 0.05 *vs.* conventional antibiotics alone.

Many bactericidal antimicrobials have been known to share a common lethal pathway that involves the generation/accumulation of hydroxyl radicals [17]. To explore this idea, we sought to determine whether any of the various CLP-19 and conventional antibiotic combinations was capable of generating hydroxyl radicals. Assay with dye30-(p-hydroxyphenyl) fluorescein (HPF), a cell-permeable fluorescence probe that selectively detects highly reactive oxygen species such as hydroxyl radicals, showed that treatment of CLP-19, ampicillin or ceftazidime alone significantly generated hydroxyl radicals by > 60% against *E. Coli*. However, levofloxacin alone only generated hydroxyl radicals by < 10%. When the combination treatments were applied the hydroxyl radicals significantly increased (*p* < 0.05) for ampicillin, ceftazidime or levofloxacin (Figure 2A). Tests against the *S. aureus* strains showed similar effects when the combination treatments were applied using the three above antibiotic agents (*p* < 0.05). Additionally, the low level of hydroxyl radicals produced by the erythromycin treatment was enhanced by CLP-19 (*p* > 0.05), which may elucidate our previous observation of only a partial synergistic effect between CLP-19 and erythromycin against S.au*reus* (Figure 2B)*.* Meanwhile, the marked increasing of hydroxyl radicals (*p* < 0.05) was also observed when CLP-19 in combination with ceftazidime against *A. baumannii* (data not shown).

### CLP-19 or CLP-19-based combination treatments induced catabolic NADH depletion

**Figure 3:**
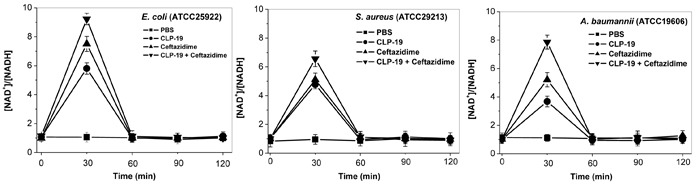
Catabolic NADH depletion induced by CLP-19 alone, ceftazidime alone or the combination treatment Bacterial cells at the mid-log phase (1×10^6^/mL) were treated with MICs of CLP-19 alone, ceftazidime alone or in combination, and then were collected by centrifugation at 13 000 rpm for 1 min at every half hour between 0 and 2 h. The NAD^+^ cycling assay was performed in 96-well plates and the OD_570_ was recorded within 10 min. NAD^+^ and NADH standards ranging between 0.0375 and 0.75 nM were used to calibrate the assay. Cells treated with PBS served as controls. Numerical data represent mean ± SD (*n* = 3).

To understand the mechanism underlying the formation of hydroxyl radicals, the depletion of NADH stimulated by CLP-19, ceftazidime and their combination was investigated by measuring the NAD^+^/NADH ratios. The results showed that CLP-19 or ceftazidime alone markedly increased the NAD^+^/NADH ratio of the various bacterial organisms tested by > 3- to 7-fold at 0.5 h after the agents were added. Notably, more than 6- to 9-fold in the NAD^+^/NADH ratios were occurred in response to treatment of CLP-19 in combination with ceftazidime at 0.5 h after the agents were added, but the NAD^+^/NADH ratio values returned to levels indistinguishable from initial values by the 1 h post-treatment time point. These changes were not observed in the untreated control group, wherein the NAD^+^/NADH ratio remained relatively consistent (Figure 3).

### CLP-19 attenuates antibiotic-induced LPS release

**Figure 4:**
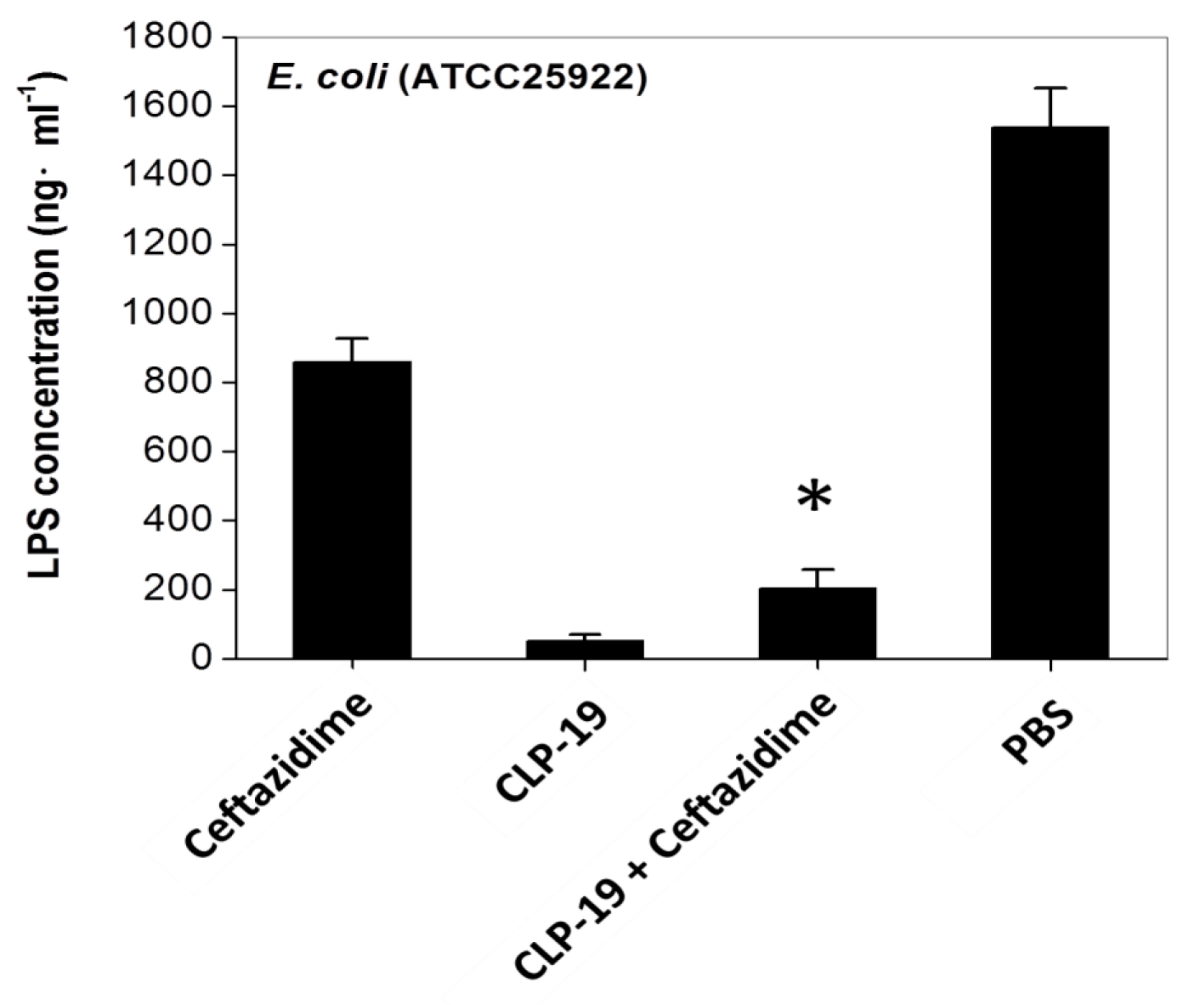
Influence of CLP-19 on antibiotic-induced LPS endotoxin release Bacterial cells at the mid-log phase (1×10^4^/mL) were treated with 2× MICs of CLP-19 alone, ceftazidime alone or in combination by incubation at 37°C for 6 h. The filtrates of cultures were serially diluted and reacted to the LAL reagent. Kinetic turbidity was measured using an ATi-321 tube reader. The PBS treated bacterial cells served as controls. Numerical data represent mean ± SD (*n* = 3). *p < 0.05 *vs.* ceftazidime.

The concentration of LPS in supernatants was measured to investigate the effect of CLP-19 on antibiotic-liberated LPS release. The CLP-19-treated cultures showed the lowest concentrations of LPS, and ceftazidime treatment led to significant LPS liberation at 2×MIC. However, when bacterial cells were treated with the combination of ceftazidime and CLP-19, the release of LPS was decreased tremendously (*p* < 0.05), and this was in contrast to the PBS-treated control cultures showing the highest concentrations of liberated LPS were found in supernatants (Figure 4).

## DISCUSSION

Drug-resistant bacterial infections have a high mortality rate and the treatment process is difficult since the conventional antibacterial drugs have reduced efficacy or are completely inefficacious. Alternative novel antibacterial agents or combination antibiotic therapy are considered to a promising strategy for overcoming this obstacle. The present study confirmed our previous finding that CLP-19 exhibits a robust antibacterial activity against Gram-positive and Gram-negative bacteria. Intriguingly, a significant synergistic effect with higher potency, faster killing kinetics and longer antibacterial duration was observed when CLP-19 was applied in combination with other bactericidal and bacteriostatic antibiotics, and these findings were in accord with other AMPs or antibacterial agents [18–21].

However, not all combinations exert synergistic effect. For instance, the bacteriostatic antibiotics were discovered to impair the AMPs-mediated bacteria killing by depolarization the transmembrane potential [22]. In our investigation, CLP-19 exhibits non-specific activity regardless of the Gram-status and produces a synergistic effect hints that CLP-19 possibly shares another killing or synergy mechanism. Previous reports showed that antibacterial agents induced highly deleterious hydroxyl radical production, suggesting oxidative stress acts as the trigger inducing bacterial cell death [17, 23]. Our further mechanism research is in agreement with these studies that CLP-19 and CLP-19-based combination significantly increase the generation of hydroxyl radicals from bacteria through transiently boosting NAD^+^ production (or depleted NADH), which are closely associated with the direct and synergistic antibacterial effect of CLP-19, rather than a consequence of bacterial killing.

Moreover, the antibiotic-induced liberation of LPS, especially by some classes of β-lactam antibiotics such as ceftazidime against Gram-negative bacilli, has been shown to be associated with rapid clinical deterioration of patients [24, 25]. Our previous work have revealed that CLP-19 in the extracellular space can bind directly to LPS and competitively inhibit formation of the LPS/LBP complex, thereby preventing the subsequent TLR4 activation and successive induction of cytokines [14]. Therefore, ceftazidime and *E. coli* were employed to set up models to detect the anti-LPS activity of CLP-19-based combination. Provocatively, our present finding demonstrates that the direct anti-LPS activity of CLP-19 contributes to relief of antibiotic-induced LPS liberation and successive fatal adverse effects.

In conclusion, our investigation showed the compelling direct antibacterial activity and synergism with bactericidal and bacteriostatic antibiotics. More importantly, treatment of CLP-19 is able to reduce other conventional antibiotic-induced liberation of LPS. We therefore revealed that CLP-19 is a potential therapeutic agent and adjuvant for treatment of bacterial infection.

## MATERIALS AND METHODS

### Preparation of peptides

The head-to-tail-looped peptide CLP-19 (CRKPTFRRLKWKIKFKFKC; molecular mass, 2511.1 Da) and S-LALF peptide (CHYRIKPTFRRLKWKYKGKFWC; molecular mass, 2945.5 Da) were synthesized by the Symphony Peptide Synthesizer (Protein Technologies, Tucson, AZ, USA) using a stepwise solid-phase peptide assembly procedure. The synthesis of peptide CLP-19 was started with an Fmoc-Lys(Boc)-Wang resin. After drying, the peptides were cleaved and purified by trifluoroacetic acid mixture solution and HPLC (Shen Zhen Hybio Engineering, Shenzhen, China) to achieve a purity of 98.4% for CLP-19 and 99.2% for S-LALF.

### Antibiotics, bacterial strains and culture conditions

Ampicillin, ceftazidime, erythromycin and levofloxacin were purchased from Sigma-Aldrich (St. Louis, MO, USA). *E. coli* (ATCC 25922), *S. aureus* (ATCC 29213), *A. baumannii* (ATCC 19606), and *P. aeruginosa* (ATCC 27853) were obtained from the American Type Culture Collection (Manassas, VA, USA). The pathogenic bacteria were grown in Difco Luria Bertani (LB) medium at 37°C.

### Antibacterial activity assay

Bacterial strains were cultured in Mueller-Hinton (MH) broth and concentration of the bacteria suspensions were adjusted to obtain standardized populations by measuring the turbidity with a SpectraMax M2e spectrophotometer (Molecular Devices, Sunnyvale, CA, USA). At mid-log phase, the bacterial strains (1×10^6^/mL) were inoculated into MH broth and the mixture (0.1 mL) was dispensed into 96-well microtiter plates. Susceptibility testing was performed by broth microdilution of the test compounds, as recommended by guidelines of the Clinical and Laboratory Standards Institute (CLSI) (2015). Briefly, after 18 h of incubation at 37°C, the MIC and growth was assayed by monitoring optical density at 620 nm (OD_620_).

### Haemolysis assay

Defibrinated horse blood (2%, Oxoid, Basingstoke, UK) and CLP-19 were co-incubated in PBS buffers at defined pH 7.4 (35 mM phosphate buffer/150 mM NaCl). Following incubation in a microplate spectrophotometerat 37°C for 4 h, the amount of hemoglobin released was spectrophotometrically measured with A_570_ readings every 2 min. The measurement was then quantified relative to positive control samples lysed with 1% sodium dodecyl sulfate, which leads to 100% haemolysis. A reduction in the A_570_ reading indicated hemolysis.

### Mammalian cell toxicity assay

Vero cells were obtained from the Department of Immunology at the Third Military Medical University (Chongqing, China) and cultured in RPMI 1640 (Invitrogen, Shanghai, China) supplemented with 10% fetal bovine serum (TBD Sciences, Tianjing, China), 100 U/mL penicillin and 100 U/mL streptomycin (Beyotime, Jiangsu, China) at 37°C under 5% CO_2_ [26, 27]. Cells were harvested by 0.05% trypsin to prepare single cell suspension, seeded in 96-well plates and cultured for 24 h prior to treatment of CLP-19. An increasing amount of CLP-19 was added and cells were incubated for another 48 h. After washing, cells were further cultured in fresh medium containing 100 mg/L neutral red for 90 min prior to a PBS wash. Then cells were treated with 200 μL of acidified isopropanol (0.33% HCl), and the viability was assessed with A_540_. A reduction in A_540_ values indicate reduced cell viability.

### Combination assay

The overall antibacterial effect of CLP-19 in combination with the conventional antibiotics was investigated by checkerboard analysis *via* readout of the FICI. FICI was calculated according to the equation: FIC_A_+ FIC_B_, where FIC_A_ was [(MIC_Drug A_ in combination)/(MIC_Drug A_ alone)] and FIC_B_ was [(MIC_Drug B_ in combination)/(MIC_Drug B_ alone)] [28, 29]. The resultant values were interpreted as follows: FICI < 0.5, synergy; 0.5 ≤ FICI < 1, partial synergy; 1 ≤ FICI < 4, additive effect or indifference; 4 ≤ FICI, antagonism [30]. A2-dimensional checkerboard with 2-fold dilutions of each agent was set up following the CLSI guidelines (2015). Growth control wells containing medium and no antibiotic agents were included in each plate. Each test was performed in triplicate.

### Antibacterial kinetics assay

Bacteria suspension at mid-log phase (1×10^6^/mL) was plated in 96-well microtiter plates and treated with CLP-19, ceftazidime, or co-treated with CLP-19 and ceftazidime. After incubation at 25°C for 5 min, 15 min, 30 min, 1 h, 3 h, 6 h and 24 h, the amounts of viable bacteria were determined by serial dilution in PBS and plating on trypticase soy agar (TSA) plates with 24 h of incubation at 37°C. The detection limit for each well was 100 CFU/mL.

### Disc diffusion assay

To assess the temporal stability of the antibacterial actions of CLP-19 alone, ceftazidime alone and the combined treatment, sterile paper discs (AA, diameter 6 mm; Whatman International Ltd, Maidstone, Kent, UK) were impregnated with MICs of each or with PBS only as a control. Discs were dried for 3 h at 25°C and placed onto the surface of a TSA plate that had been seeded with 100 μL of *E. coli* suspension (1×10^8^/mL; spread to achieve a semi-confluent lawn of growth) and allowed to dry for 3 h at 37°C. The plates were then incubated for 5 days at 37°C and inspected every 24 h for the appearance of clear zones around each disc, which were measured with a ruler. Growth inhibition was calculated according to the equation: [(total clear zone area)-(area of the disc)].

### Hydroxyl radical formation assay

Bacteria (1×10^6^/mL) were treated with MICs of CLP-19, ampicillin, ceftazidime, erythromycin, levofloxacin or CLP-19 in combination with each conventional antibiotic, and PBS served as controls. All experimental groups were incubated at 37°C for 2 h. 5 mM of fluorescent reporter dye30-(p-hydroxyphenyl) fluorescein (HPF) (Invitrogen) was subsequently added. The fluorescence intensity of HPF was measured by a spectrofluorophotometer at 490 nm excitation and 515 nm emission wavelengths. The percentage of hydroxyl radical formation was calculated based on an equation: [(OD_490_ of well treated with antibacterial agent)-(OD_490_ of non-treated control)]/(OD_490_ of non-treated control)×100.

### NAD^+^, NADH extraction

Dinucleotide extraction and the cycling assay were performed, as previously described [31]. Bacteria at the mid-log phase (1×10^6^/mL) were centrifuged at 13000 rpm for 5 min and resuspended in 1 mL of LB. For NAD^+^ and NADH extraction, bacteria samples were collected by centrifugation (13000 rpm for 1 min) at every half hour between 0 and 2 h after addition of MICs of CLP-19, ceftazidime, or in combination. The supernatant was removed and the pellets were frozen immediately in a dry ice-ethanol bath and stored at −80°C until all samples had been collected. Analysis of the ice-cold pellets was initiated by adding a 75 μL of 0.2 M NaOH (for NADH extraction) or 75 μL of 0.2 M HCl (for NAD^+^ extraction), after which the samples were heated for 10 min at 100°C and then centrifuged at 10000 rpm for 5 min. The NAD^+^/NADH-containing supernatants were transferred to fresh tubes and stored in the dark on ice until use in the cycling assay.

The NAD^+^ cycling assay was performed in 96-well plates. The respective reaction mixtures contained 16 μL of 1.0 M bicine (pH 8.0) (Sigma-Aldrich), 40 μL of sample extract, 40 μL of neutralizing buffer (0.1M HCl for NADH or 0.1 M NaOH for NAD^+^), 16 μL of 100% ethanol, and 30 μL of 40mM EDTA (pH 8.0). When the reaction mixtures were aliquoted in the wells, 16 μL phenazine ethosulfate (PES) (Sigma-Aldrich) and 16 μL 3- [4,5-dimethylthiazol-2-yl]-2,5-diphenyltetrazolium bromide (MTT) (Sigma-Aldrich) were added and the plates were incubated for 3 min at 30°C. Then, 3.2 μL of alcohol dehydrogenase (500 U/mL; Sigma-Aldrich) in bicine buffer (pH 8.0) were added to the reaction mixture to initiate the reaction and the increase in absorbance at 570 nm over the next 10 min was recorded. The rate of MTT reduction is proportional to the concentration of NAD^+^ or NADH in the sample, and NAD^+^ and NADH standards (range:0.0375 and 0.75 nM; Sigma-Aldrich) were used to calibrate the assay.

### Endotoxin release studies

Bacteria at the mid-log phase (1×10^4^/mL) were treated with 2×MICs of CLP-19, ceftazidime, or in combination for 6 h at 37°C. Bacteria treated with PBS served as controls. The samples were filtered through a pyrogen-free 0.2-pm pore polysulphone filter (Acrodisc; Gelman Scientific, Northampton, UK) and the filtrates were stored immediately at −70°C until use. For analysis, the filtrates were thawed at room temperature, serially diluted with pyrogen-free water (range: 10^2^- to 10^4^-fold) and reacted with Limulus amebocytelysate (LAL) reagent (1:1 ratio). Kinetic turbidity was measured using an ATi-321 tube reader (Lab Kinetics, Somerset, UK).

### Statistical analysis

Data are expressed as the mean of at least three independent experiments ± standard deviation. Statistical significance was determined by paired Student *t* test if compared only two groups or one-way ANOVA followed by what test if analyzing more than two groups. Differences were considered to be statistically significant at *P* < 0.05 [32].
